# Low phosphorus induces differential metabolic responses in eucalyptus species improving nutrient use efficiency

**DOI:** 10.3389/fpls.2022.989827

**Published:** 2022-09-15

**Authors:** Franklin Magnum de Oliveira Silva, Rafaela Gageti Bulgarelli, Umarah Mubeen, Camila Caldana, Sara Adrian L. Andrade, Paulo Mazzafera

**Affiliations:** ^1^Department of Plant Biology, Institute of Biology, State University of Campinas, Campinas, Brazil; ^2^Department of Molecular Physiology, Max Planck Institute of Molecular Plant Physiology, Potsdam, Germany; ^3^Department of Crop Production, Luiz de Queiroz College of Agriculture, University of São Paulo, São Paulo, Brazil

**Keywords:** plant nutrition, metabolome, lipidome, phosphorus use efficiency (PUE), primary metabolites

## Abstract

Phosphorus (P) is a vital nutrient for plant growth. P availability is generally low in soils, and plant responses to low P availability need to be better understood. In a previous study, we studied the growth and physiological responses of 24 species to low P availability in the soil and verified of eucalypts, five (*Eucalyptus acmenoides*, *E. grandis*, *E. globulus*, *E. tereticornis*, and *Corymbia maculata*) contrasted regarding their efficiency and responsiveness to soil P availability. Here, we obtained the metabolomic and lipidomic profile of leaves, stems, and roots from these species growing under low (4.5 mg dm^–3^) and sufficient (10.8 mg dm^–3^) P in the soil. Disregarding the level of P in the soils, P allocation was always higher in the stems. However, when grown in the P-sufficient soil, the stems steadily were the largest compartment of the total plant P. Under low P, the relative contents of primary metabolites, such as amino acids, TCA cycle intermediates, organic acids and carbohydrates, changed differently depending on the species. Additionally, phosphorylated metabolites showed enhanced turnover or reductions. While photosynthetic efficiencies were not related to higher biomass production, *A*/*C*i curves showed that reduced P availability increased the eucalypt species’ *Vcmax, Jmax* and *photosynthetic P-use efficiency*. Plants of *E. acmenoides* increased galactolipids and sulfolipids in leaves more than other eucalypt species, suggesting that lipid remodelling can be a strategy to cope with the P shortage in this species. Our findings offer insights to understand genotypic efficiency among eucalypt species to accommodate primary metabolism under low soil P availability and eventually be used as biochemical markers for breeding programs.

## Introduction

Phosphorus (P) limits the productivity of plants in many terrestrial ecosystems (Batjes, 1997), and it is often the first or second nutrient limiting aboveground net primary productivity of forests (Gonçalves et al., 2008; Plassard and Dell, 2010; Lang et al., 2016). Besides being an integral component of membrane lipids and nucleic acids, P is a component of the ATP molecule that confers to this nutrient a central role in almost all aspects of plant metabolism (Warren, 2011).

Plants respond to P limitation in several ways, including changes in the architecture of the root system and alterations of metabolic processes (Jones et al., 2018). Morphological and physiological changes of the root system in response to P deficiency are often related to developmental strategies which allow a more efficient exploration and mining of soil P (Niu et al., 2013) as shown in eucalyptus (Bichara et al., 2021). Plants may increase the root-shoot ratio by reducing and enhancing shoot and root growth, respectively (Niu et al., 2013; Lambers et al., 2015a,b; Tian and Liao, 2015) or yet establishing beneficial mutualistic associations with mycorrhizal fungi (Lambers et al., 2008). Root growth may be accompanied by an increased release of organic acids and enzymes to improve P availability (Lambers et al., 2015a,b). In addition, to improve the efficiency of internal P use, plants such as barley, may increase their internal amino- and organic acid levels (Huang et al., 2008), accumulate di- and tri-saccharides, reduce the content of phosphorylated sugars as observed in maize, wheat and arabidopsis (Huang et al., 2008; Ganie et al., 2015; Pant et al., 2015a; Nguyen et al., 2019). Recently, the metabolome analysis of P-stressed apple seedlings revealed that amino acids, organic acids and flavonoids levels increased (Sun et al., 2021). However, P deficiency had little effect on primary metabolites, such as carbohydrates, organic acids and amino acids in *E. globulus* (Warren, 2011). On the other hand, P fertilisation altered just a small portion of polar metabolites in leaves of *Pinus elliottii* × *P. caribaea* (Guo et al., 2022).

Plants can also replace membrane phospholipids with sulfolipids and galactolipids, thus, reducing P requirements (Lambers et al., 2012; Yuzawa et al., 2012; Okazaki et al., 2013; Shimojima et al., 2013; Lambers, 2022). *Fagus sylvatica* avoids the seasonal loss of P by degrading phospholipids in senescent leaves and exporting P to the wood tissues, where P is used for the biosynthesis of new phospholipids and N-acetyl-D-glucosamine-6-phosphate (Netzer et al., 2018).

Thus, under P deficient conditions, plants may adjust growth and development by changing carbon (C) allocation among organs and organelles to tolerate the stress better and increase the efficiency of main metabolic processes (Morales et al., 2020). However, until now, it remains unclear how P deficiency affects the metabolism of evergreen trees, even though the complete elucidation of this strategy could help further understand the physiology of plant P acquisition (Müller et al., 2015).

Eucalypt plantations frequently cope with P limitation, showing visual symptoms of P deficiency and high responses to P-fertilisation (Santana et al., 2002; Thomas et al., 2006). Recently, we used physiological and nutritional parameters to evaluate the ability of 24 species of eucalypts to grow under low and sufficient soil P conditions (Bulgarelli et al., 2019). Considering that plants may be efficient to grow on low P and/or responsive to P inputs we may classify them into four groups: efficient and responsive, non-efficient and non-responsive, non-efficient and responsive, and efficient and non-responsive. Most of the 24 species of eucalyptus were distributed between two groups: (i) non-efficient in the use of P and responsive to P addition and (ii) efficient and non-responsive. Here, we studied changes in the metabolomic and lipidomic profile of leaves, stems, and roots of plants of five species (*Eucalyptus acmenoides*, *E. globulus, E. grandis, E. tereticornis, and Corymbia maculata*) belonging to these two groups, growing under sufficient and low P soil availability. We aimed to evaluate how efficiency and responsiveness might be related to these changes. *E. grandis*, which genome is available (Myburg et al., 2014), was included in the study as a reference species.

## Materials and methods

### Plant growth and sampling

The experiment was conducted in a greenhouse between September 2018 and April 2019, at Campinas (22°49′10.38′′S 47°04′12.88′′W), São Paulo, Brazil. The experimental design was a 5 × 2 factorial, i.e., five eucalypt species and two soil P levels, with five replicates of each species for each soil P level. The species were: *Corymbia maculata*, *Eucalyptus acmenoides*, *E. globulus*, *E. grandis* and *E. tereticornis*. The soil used here was a clay ferrosol (IUSS Working Group WRB, 2015), and P is referred to as the soil resin extractable-P. The soil analysis is shown in Supplementary Table S1. Before mixing 1:1 (v/v) with water-washed sand, the soil was corrected for nutrients as recommended for forest species at a medium-fertility level in São Paulo, Brazil (Raij et al., 1997). The soil P level was not correct in one treatment (4.1 mg dm^–3^ available P) and corrected (12.9 mg dm^–3^ available P) in another, and they are referred to from now on as low P and sufficient P, respectively. We amended the low P soil with potassium phosphate (KH_2_PO_4_) to reach the P level in sufficient soil.

Seeds of the five species were germinated in seedbeds with a commercial substrate mixture (Genesolo, Genfertil), and after 2 months the seedlings were transplanted to 7 L pots containing the mixture of soil and sand described previously. They were kept in a greenhouse for a period of 9 months. Irrigation was carried once a day. During the experiment, the seedlings received five applications of N (125 mg each application), using NH_4_NO_3_ or KNO_3_.

Leaf, stem, and root samples of 8-month-old plants were harvested separately and immediately frozen in liquid nitrogen and stored at −80°C until further analysis. The roots were collected after washing out the substrate in running tap water.

### P determination

The concentrations of P in leaves, stems and roots were determined by inductively coupled plasma with optical emission spectrometry (ICP-OES, Varian Vista MPX, Palo Alto, CA, United States) in nitro-perchloric acid digested samples (Zasoski and Burau, 1977). Photosynthetic P-use efficiency (PPUE, μmol CO_2_ mol P^–1^ s^–1^) and P utilisation efficiency (PU_*t*_E, g DW g^–1^ P) were calculated according to Hammond et al. (2009).

### Leaf gas exchange and chlorophyll a fluorescence

Leaf gas exchange and chlorophyll *a* fluorescence analyses were performed simultaneously using an open-flow infrared gas exchange analyser system equipped with an integrated fluorescence chamber (IRGA, LI-6400XT). Photosynthetic light-response curves, the rate of photosynthesis (*A*) as a function of photosynthetic photon flux density (PPFD), were obtained using atmospheric CO_2_ concentration (*C*_*a*_), which was 400 μmol CO_2_ mol^–1^, and the plants were exposed to a range of PPFD in a stepwise sequence of 1,000, 2,000, 1,500, 1,000, 800, 600, 300, 150, 100 and 0 μmol photons m^–2^ s^–1^. Variables derived from the *A*/PPFD curves were estimated from the light response curve adjustments by the non-rectangular hyperbolic model (Caemmerer, 2000). Measurements for CO_2_ response curves, *A* as a function of intercellular CO_2_ concentrations (*C*_*i*_) (A/*C*_*i*_), were taken at a light saturation point of 1,000 μmol photons m^–2^ s^–1^, 25°C at *C*_*a*_ of 400 μmol CO_2_ mol^–1^ and once the steady-state was reached, the *C*_*a*_ was decreased to 300, 200, 100, 50, and 25 μmol CO_2_ mol^–1^. Then, *C*_*a*_ was increased to 400, 500, 700, 1,000 and 1,200 μmol CO_2_ mol^–1^. Curves were obtained using the second terminal leaflet of the third fully expanded leaf. The data were subjected to an analysis of variance (ANOVA), and the group of means of eucalypt species were compared with subtropical specie model *E. grandis* by the Scott-Knott test at *P* ≤ 0.05.

### Primary metabolites and lipid extraction

Leaves, stems and roots of five independent biological replicates were ground to a fine powder, and 30 mg (dry weight ± 1% tolerance) was extracted with 1 ml of a pre-cooled (∼15°C) mixture of methyl *tert*-butyl ether (MTBE; 3:1:1, v/v/v) as described previously by Giavalisco et al. (2011) and Salem et al. (2016). Then, the samples were thoroughly vortexed for 1 min and incubated on an orbital shaker (100 rpm) for 45 min at 4°C, followed by a 15 min sonication step. For phase separation, a volume of 500 μl of water:methanol (3:1, v/v) was added to each vial, and the samples were again thoroughly vortexed for 1 min. The samples were centrifuged at 20,000 × *g* for 5 min at 4°C, and 300 μl of the upper apolar MTBE phase containing the lipids were transferred to a 1.5 ml vial and vacuum-dried. The remaining MTBE phase was entirely removed from the extract, and two independent aliquots of 150 μl of the lower polar fraction were transferred to a new vial and concentrated to dryness in a vacuum concentrator for subsequent analysis by gas chromatography (GC).

### Lipid profiling

The lipids extracted from the previous step were solubilised in 300 μl of a mixture of acetonitrile:isopropanol (7:3 v/v), vortexed and incubated 10 min in an ultrasonication bath. The samples were centrifuged for 5 min at 10,000 × *g*, and 100 μl aliquots were transferred into glass vials for further analysis (Salem et al., 2016). Lipids in 2 μl injection samples were separated on a reversed-phase Bridged Ethyl Hybrid (BEH) C8 column (100 mm × 2.1 mm containing 1.7 μm diameter particles, Waters, Manchester, United Kingdom) using a Waters Acquity UPLC system (Waters, Manchester, United Kingdom) (Hummel et al., 2011). The UPLC–FT–MS raw chromatograms were analysed and processed either by using Xcalibur (Version 2.10, Thermo-Fisher, Bremen, Germany) or ToxID (Version 2.1.1, Thermo-Fisher) as described in Hummel et al. (2011).

### Primary metabolite profiling

Dried aliquots of the polar phase of the previously described extraction were solubilised in 150 μl methoxyamine-hydrochloride/pyridine solution for methoxymization of carbonyl groups, followed by heating at 37°C for 90 min. The samples were derivatised with *N*-methyl-*N*-(trimethylsilyl)trifluoroacetamide (MSTFA) for 30 min at 37°C. An aliquot of 1 μl of the derivatised sample was analysed by GC-MS, according to da Silva et al. (2018). We used the softwares Chroma TOF^®^ 4.2 (Leco, St. Joseph, MI, United States) and TagFinder 4.0 (Luedemann et al., 2012) to analyse the chromatograms and mass spectra. Analytes were manually identified using TagFinder, by comparing them to the reference library mass spectra and retention time indices in the Golm Metabolome Database (Kopka et al., 2005). The amounts of metabolites were expressed as relative abundance, calculated by the normalisation of the sorbitol signal intensity, which was added as an internal standard, and later by the dry weight of the initial plant material.

### Statistical analysis

The overall effects of soil P availability and eucalypt species on the photosynthesis parameters and metabolic levels were evaluated using a two-factor analysis of variance (two-way ANOVA). The means were compared by Scott-Knott test at *P* < 0.05, using the Rbio software (Bhering, 2017). Graphical representations were performed using SigmaPlot 11.0^®^ software (Systat Software Inc.). In order to reduce the dimensionality of the data set, MetaboAnalyst 4.0 (Chong et al., 2018) and Minitab^®^ 17 were employed for multivariate data analysis, including a principal component analysis (PCA) and heatmap matrices.

## Results

### Phosphorus in plants

In most species, soil P availability affected the concentrations of one or more of the other analysed nutrients (Table 1). When grown in low P conditions, *E. acmenoides, E. grandis and C. maculata* presented significantly higher P concentrations in leaves when compared to sufficient P. Whereas for *E. globulus* and *E. tereticornis* foliar P concentrations were similar under low and sufficient soil P availability. Stem (0.7 to 1.09 g kg^–1^) and root (0.44 to 0.77 g kg^–1^) P concentrations were higher in plants of *E. tereticornis* grown with sufficient P than in those with low P. It is worth highlighting that the sufficient soil P availability positively and linearly influenced the accumulation of P in the stems of all eucalypt species, except in *E. globulus* (Table 1).

**TABLE 1:** Concentrations of phosphorus in the roots, stems and leaves of five eucalypt species cultivated in soil with low (Low P) and sufficient (Suf P) phosphorus availability.

		P (g kg^–1^)											
Species	Phosphorus	Roots				Stems				Leaves			
*C. maculata*	Low P	0.59	±	0.10	**a**	0.42	±	0.01	**a**	0.77	±	0.02	**a**
*E. acmenoides*	Low P	0.50	±	0.04	**b**	0.56	±	0.06	**a**	0.57	±	0.02	**b**
*E. grandis*	Low P	0.43	±	0.03	**b**	0.51	±	0.07	**a**	0.58	±	0.06	**b**
*E. globulus*	Low P	0.65	±	0.04	**a**	0.70	±	0.06	**a**	0.91	±	0.02	**a**
*E. tereticornis*	Low P	0.44	±	0.01	**b**	0.70	±	0.17	**a**	0.74	±	0.09	**a**
*C. maculata*	Suf P	0.61	±	0.04	**b**	1.03	±	0.11	a*	0.96	±	0.03	a*
*E. acmenoides*	Suf P	0.43	±	0.03	**c**	1.18	±	0.09	a*	0.88	±	0.02	a*
*E. grandis*	Suf P	0.62	±	0.03	b*	1.20	±	0.21	a*	0.78	±	0.10	b*
*E. globulus*	Suf P	0.77	±	0.02	a*	0.76	±	0.09	**a**	0.89	±	0.04	**a**
*E. tereticornis*	Suf P	0.77	±	0.07	a*	1.09	±	0.14	a*	0.72	±	0.02	**b**

Means followed by the same letter indicate no statistical difference by the Scott-Knott test (P > 0.05) among species in each treatment and tissue. Asterisks indicate a statistical difference between P treatments for each species and tissue (Scott-Knott test, P < 0.05).

### Biomass production and P use efficiency

**FIGURE 1:**
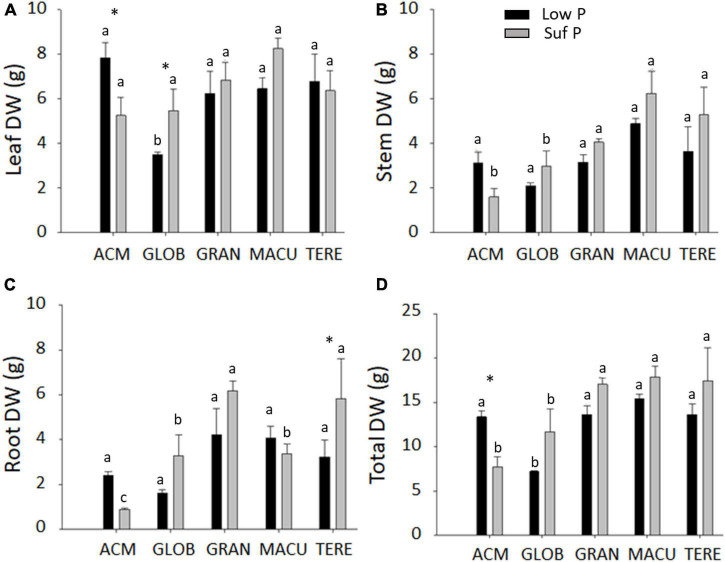
Leaf **(A)**, stem **(B)**, root **(C)**, and total **(D)** dry weight of five eucalypt species cultivated in soil with low (Low P) and sufficient (Suf P) availability of phosphorus. *E. acmenoides* = ACM, *E. globulus* = GLOB, *E. grandis* = GRAN, *C. maculata* = MACU, *E. tereticornis* = TERE. Means with the same letter indicate no statistical difference among species in each P treatment by Scott-Knott test (*P* < 0.05). Asterisks indicate statistically different between P treatments within the same species by the Scott-Knott test (*P* < 0.05).

In general, plant growth (leaf, stem and root mass production) was higher in the sufficient P treatment than low P (Figure 1). An exception was *E. acmenoides*, which showed lower biomass under sufficient P, and statistically different for leaf and total dry mass, but not for stem mass (Figures 1A,B,D). When grown in low P, *E. globulus* presented the lowest mass production for leaf, stem and root, reflected in the total plant biomass (Figure 1D). Interestingly, *E. acmenoides* plants in the presence of high P availability contained about two times lower root mass than other eucalypt species (Figure 1C).

The calculated P utilisation efficiency (PUtE) and photosynthetic P-use efficiency (PPUE) showed that *C. maculata* and *E. tereticornis* presented the lowest PUtE (382.64 and 558.96 g DM g^–1^ P, respectively) and *E. acmenoides* the highest (13093.77 g DM g^–1^ P) (Supplementary Table S2). *E. tereticornis* presented the highest and *E. maculata* the lowest PPUE, in both P availability (Supplementary Table S2). *E. acmenoides and E. tereticornis* were the only species to present low PPUE when growing in low P, which might be explained by the high and low photosynthetic CO_2_ assimilation capacity observed in these species, respectively (Supplementary Table S2).

### Effects of soil P availability on photosynthesis

**FIGURE 2:**
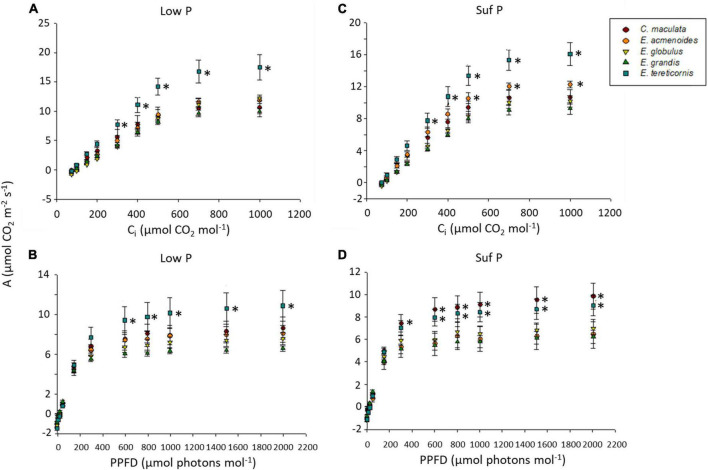
Curves of CO_2_-assimilation rates of five eucalypt species cultivated in soil with low (Low P) or sufficient (Suf P) phosphorus availability. CO_2_-assimilation rate (*A*) as a function of intercellular CO_2_ concentrations (*C*_i_) **(A,C)** and CO_2_-assimilation rate (*A*) as a function of photosynthetic photon flux density (PPFD) **(B,D)**. Asterisks on the right side of symbols indicate a statistical difference between the species (symbol) and *E. grandis*, according to the Scott Knott test (*P* < 0.05).

Because P plays an essential role in photosynthesis and the partitioning of photosynthates, we analysed *A*/*Ci* and *A*/PPFD response curves in the five eucalypt species growing under low and sufficient P (Figures 2A–D). Under low P, the photosynthetic rate (*A*) varied between species means of 6 and 11 μmol m^–2^ s^–1^, stomatal conductance (g_s_) between 0.17 and 0.27 mol m^–2^ s^–1^ and transpiration (Trmmol) between 2.43 and 3.88 mmol H_2_O m^–2^s^–1^ (Supplementary Table S4).

The soil P availability led to a significant increase of electron transport rate (ETR), maximum carboxylation velocity based on C_i_ (V_*cmax*__*C*_i_), and maximum capacity for electron transport rate based on C_i_ (J_*max*__*C*_i_) in *E. tereticornis* compared to other species (Supplementary Table S3). Moreover, independently of soil P availability, *E. tereticornis* had the highest light saturation point, which was approximately 1,000 μmol m^–2^ s^–1^, while in the other species, it was 800 μmol m^–2^ s^–1^, indicating interspecific variation of the photosynthetic capacity. Surprisingly, the overall response concerning the photosynthetic characterisation of the species under varied atmospheric CO_2_ was similar between the two P levels within each species (upper case letters in Supplementary Table S3). On the other hand, we found differences among species for each P availability (lower case letters in Supplementary Table S4).

### Changes in the metabolome and lipidome caused by P availability

**FIGURE 3:**
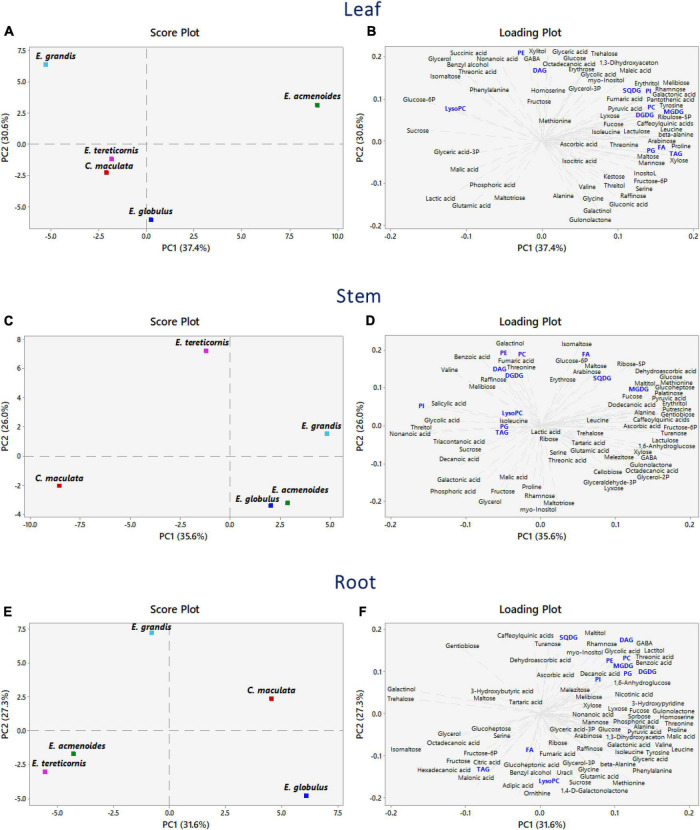
Principal component analysis combining metabolomic and lipidomic profiles from five eucalypt species cultivated in soil with low (Low P) and sufficient (Suf P) availability of phosphorus. The analysis was performed by the changes of log2 ratios (Low P/Suf P) metabolic contents in leaves, stems and roots, individually. Numbers in parentheses indicate the percentual variation of the first (PC1) and the second (PC2) principal components. **(A,C,E)** Show the scores and **(B,D,F)** show the loading plots. Lipids are in blue. Monogalactosyldiacylglycerol (MGDG), digalactosyldiacylglycerol (DGDG), sulfoquinovosyldiacylglycerol (SQDG), fatty acid (FA), phosphatidylinositol (PI), phosphatidylglycerol (PG), phosphatidylcholine (PC), phosphatidylethanolamine (PE), lysophosphatidylcholine (LysoPC), triacylglycerol (TAG), and diacylglycerol (DAG).

We implemented a PCA analysis to discriminate the main effects of the P availability on the dataset obtained from the metabolomic and lipidomic profiles (Figure 3). In leaves, the first and second components accounted for 37.4 and 30.6% of the total variation, respectively. The PCA score plots showed a clear separation of *E. acmenoides* from the other four species along the PC1 axis, thus suggesting that this species has a distinct metabolic response to P availability (Figure 3A and Supplementary Table S4). Overall, changes in the amino acid levels influenced this separation, mainly proline, β-alanine, tyrosine and leucine, suggesting that these metabolites may have a role in the response of *E. acmenoides* plants to low P availability. It is worth mentioning that the majority of the lipid classes increased in *E. acmenoides*. MGDG, DGDG, and TAG lipids were the main classes that contributed to this separation (Figure 3B).

The PCA scores revealed that stems had a characteristic metabolic profile in *C. maculata* and *E tereticornis* (Figure 3C), as PC1 and PC2 explained 35.6 and 26% of the total variance, respectively. At PC1, decanoic acid, nonanoic acid and valine, both intermediates of the fatty acid biosynthetic pathway, were closely related with *E. maculata* (Figure 3D and Supplementary Table S4). The PC2 variation separated *E. tereticornis* from the other species. Among various traits in PC2, galactinol and isomaltose and three classes of lipids (PE, PA and FA) explained the maximum variation. Two intermediates of the oxidative-pentose-phosphate pathway (glucose-6P and ribose-5P) were grouped to *E. tereticornis*.

The first two components explained 58.9% of the total variance in roots (Figure 3E). Opposite to leaves, *C. maculata* and *E. acmenoides* were not grouped. According to PC1 variation (31.6%), changes in the amino acid levels, such as threonine, leucine, homoserine, proline and valine, were responsible for this separation (Figure 3F and Supplementary Table S4). Hexadecanoic and octadecanoic acids, and the saccharides maltose, fructose, gentiobiose, glycerol, isomaltose, galactinol and trehalose explained the grouping of *E. tereticornis* and *E. acmenoides* (Figure 3F and Supplementary Table S4). PC2 variation revealed that most lipid classes were grouped with *C. maculata*, unlike previously observed in leaves. Overall, galactolipids and sulfolipids, such as SQDG, MGDG and DGDG, and phospholipids (e.g., PC, PE) explained the highest percentage of the total variability found in the PC2 (Figure 3F and Supplementary Table S4).

### Changes in the primary metabolites profile in response to low P

We identified 66, 75, and 66 primary metabolites in the leaves, roots and stems, respectively (Supplementary Table S5). The levels of sugars, sugar-phosphates, organic acids, polyols and amino acids were obtained by calculating a response ratio (Low P/Suf P) of the average concentration of metabolites.

**FIGURE 4:**
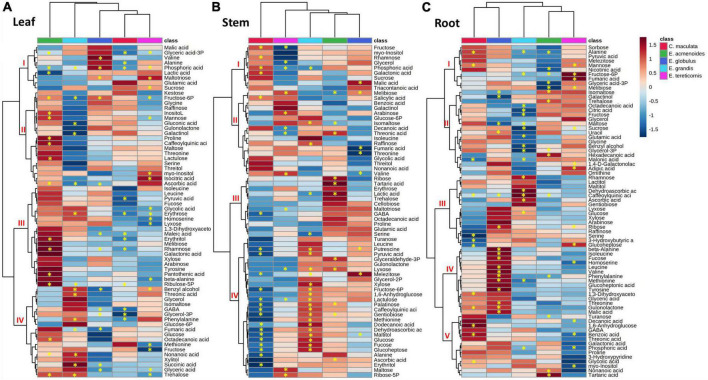
Hierarchical cluster analysis of changes in primary metabolite levels of five eucalypt species cultivated in soil with low (Low P) and sufficient (Suf P) availability of phosphorus. Heat map representing the changes of log2 ratios (Low P/Suf P) metabolic contents in leaf **(A)**, stem **(B)**, and root **(C)** individually. Regions of blue or red indicate the metabolite level was decreased or increased, respectively. The clusters were generated in MetaboAnalyst using squared Euclidean distance and complete linkage. Asterisks indicate statistically significant differences (*P* < 0.05) by Student’s *t*-test between P treatments.

A heat map prepared to facilitate data interpretation identified two main clusters within eucalypts species and three additional metabolite clusters at most (Figure 4). In the leaves, the results indicated that *E. acmenoides* and *E. grandis* were separated from *E. globulus, C. maculata and E tereticornis* (Figure 4A). Furthermore, hierarchical clustering from metabolites showed a clear difference between *E. acmenoides* and the other species. Based on clusters II and IV, ten metabolites significantly increased in leaves of *E. acmenoides* under low P; most were sugars (fructose 6-P, mannose, inositol, lactulose, erythrose, rhamnose and ribulose-5P). On the other hand, metabolites in cluster IV generally increased in *E. grandis* and decreased in the other four species. The exception was fumaric acid, which decreased in *E. grandis* and *E. globulus* in response to low P (Figure 4A).

The leaf metabolite analyses also revealed significant alterations in tricarboxylic acid (TCA) cycle intermediates. Under low P, succinic acid increased in leaves of *E. grandis* and decreased in *E. globulus*, and fumaric acid increased in the leaves of *E. globulus* and *E. grandis* (Figure 4A). Concerning the amino acids, phenylalanine increased in leaves of *E. grandis and E. globulus*, and the non-protein amino acid γ-aminobutyric acid (GABA) decreased significantly in leaves of *C. maculata and E. globulus* (Figure 4A).

Like that of leaves, the metabolite profile of the stems of *C. maculata* and *E. tereticornis* was separated from those of *E. acmenoides* and *E. grandis* (Figure 4B). Among the five species, most of the significant changes in stem metabolites in response to P availability were observed in *E. maculata* and *E. grandis*. The concentration of fructose-6P increased in the stem of *E. acmenoides* and *E. grandis* under low P. However, similar to leaves under low P, the level of this metabolite decreased in *C. maculata*. Also, main sugars, such as glucose, fructose and sucrose, were significantly affected by soil P availability. For example, *C. maculata* stems displayed decreased and increased levels of glucose and fructose under low P, respectively, while *E. grandis* has increased levels of glucose, and *E. tereticornis* showed a decrease in fructose (Figure 4B).

Regarding the TCA cycle intermediates, fumaric acid levels decreased significantly in the stem of *E. globulus*, while malic acid increased for this species under low P. As observed in leaves, GABA also showed a significant decrease in the stem of *C. maculata.* Interestingly, the pyruvate, a key metabolite of the energy metabolism, which can enter the TCA cycle or serve as the starting point for synthesising fatty acids, increased significantly in *E. globulus* under low P while decreasing in *C. maculata.* Also, valine, an intermediate of the fatty acid biosynthetic pathway, significantly reduced in *E. globulus* and increased in *E. tereticornis* (Figure 4B).

In roots, unlike leaves and stems, metabolite clustering separated *E. tereticornis* from *C. maculata* (Figure 4C). Several changes were observed in the levels of the amino acids in response to P limitation, mainly in plants of *E. globulus*, which showed a significant increase in alanine, valine, isoleucine and leucine, which are intermediates of the fatty acid biosynthesis pathway. Additionally, the concentration of intermediates of the shikimate pathway, such as phenylalanine and tyrosine, increased significantly in the roots of *E. globulus.* Other amino acids that exhibited a significant increase in the roots of this species under low P were β-alanine, valine, threonine and methionine (Figure 4C). Sugars were also significantly affected by soil P availability. Fructose and sucrose decreased in *E. grandis* roots under low P, while glucose, fucose and ribose increased. Concerning TCA cycle intermediates, low P reduced citrate levels in *E. grandis* roots compared to the other species. Other TCA intermediates, such as fumaric and malic acids, increased in the roots of *E. tereticornis* and *E. globulus*. Interestingly, 3-phosphoglyceric acid, a key metabolic intermediate in glycolysis and the Calvin cycle, decreased in *E. acmenoides* roots in response to low P (Figure 4C).

### Changes in the lipids profile in response to low P

**FIGURE 5:**
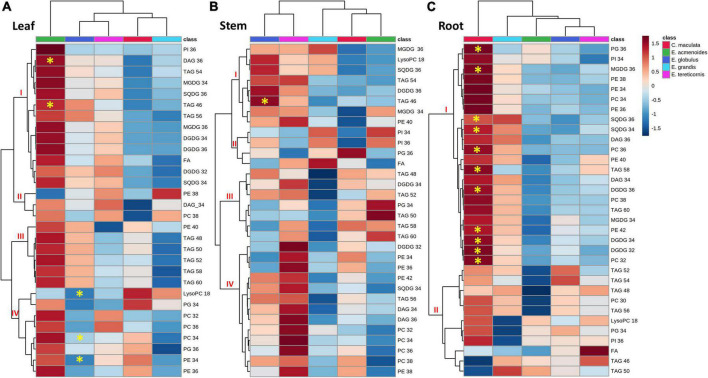
Hierarchical cluster analysis of changes in lipid profiles from five eucalypt species cultivated in soil with low (Low P) and sufficient (Suf P) availability of phosphorus. Heat map representing the changes of log2 ratios (Low P/Suf P) lipid content in leaf **(A)**, stem **(B)**, and root **(C)** individually. Regions of blue or red indicate the metabolite level was decreased or increased, respectively. The clusters were generated in MetaboAnalyst using squared Euclidean distance and complete linkage. The clusters were generated in MetaboAnalyst using squared Euclidean distance and complete linkage. Asterisks indicate statistically significant differences (*P* < 0.05) by Student’s *t*-test between treatments. MGDG, monogalactosyldiacylglycerol; DGDG, digalactosyldiacylglycerol; SQDG, sulfoquinovosyldiacylglycerol; FA, fatty acid; PI, phosphatidylinositol; PG, phosphatidylglycerol; PC, phosphatidylcholine; PE, phosphatidylethanolamine; LysoPC, lysophosphatidylcholine; LysoPA, lysophosphatidic acid; TAG, triacylglycerol; DAG, diacylglycerol.

To study possible shifts between membrane phospholipids and non-phosphorus glycerolipids, such as SQDG, MGDG and DGDG, we carried out the lipidomic analysis in leaves, stems and roots (Supplementary Table S6). We identified 172 lipid species with several chemical variants for the same main compound, and thus, classified in thirty individual lipids (for instance, DAG 34:0, DAG 34:1, DAG34:2 were classified as DAG 34), which were distributed in eleven lipid classes (Figure 5). The lipid profile of leaves of *E. acmenoides* was separated from that of the other species (Figure 5A). In general, most lipids tended to increase under low P in this species, being the exceptions LysoPC:18 and PE:18, which decreased (Figure 5A). Nevertheless, only TAG:36 and DAG:46 had a statistically significant increase. Furthermore, three groups of phospholipids from cluster IV (LysoPC:18, PC:34 and PE:34) decreased significantly in leaves of *E. globulus* under low P (Figure 5A).

In the stems, the groups of galactolipids (MGDG:36, MGDG:34 and DGDG:36), sulfolipids (SQDG:36 and SQDG:34) and triacylglycerols (TAG:46, TAG:54 and TAG:56) tended to increase in *E. globulus* and *E. tereticornis*, but only TAG:46 increased significantly (Figure 5B). In roots, the variation in response to low P was more pronounced in *C. maculata* than in the other species (Figure 5C). Under low P, 11 groups of lipids were significantly higher in *C. maculata*, and most of them were digalactosyldiacylglycerols (DGDG:32, DGDG:34 and DGDG:36), sulfoquinovosyl diacylglycerols, (SQDG:36 and SQDG:34) and phospholipids (PG:36, PC:36, PE:42, and PC:32). Nevertheless, the content of most of these lipids decreased in the roots of the other species or displayed a slight increase (Figure 5C).

## Discussion

In this work, five species of eucalypts were grown on low and sufficient soil P and compared regarding physiological and biochemical traits. We aim to understand the metabolic changes of these species regarding their ability to grow under low P availability (efficiency) and responsiveness to P supply. When grown in the P-sufficient soil, the stems steadily became the seedlings compartment with the largest amount of P, followed by leaves and roots. Dell et al. (1987) and Weggier et al. (2008) reported that eucalypt seedlings supplied with P accumulated this nutrient in the stem and bark, although some reports have shown higher accumulation in the leaves (Koutika et al., 2016). Additionally, previous studies have shown that nutrient concentrations changes in the eucalyptus tissues depending on the dimension and/or the age of the tree (Bouvet et al., 2013), with reductions of stem P concentration with plant age (Dell et al., 1987). Mature plants certainly accumulate more biomass in the stem and branches, suggesting that stems may act as the primary site for P storage in eucalypts during certain periods of growth, as previously reported for other species of evergreens (Weggier et al., 2008; Rennenberg and Herschbach, 2013). In stems of P-sensitive *E. marginata*, most P was stored in bark (Dell et al., 1987). In eucalyptus, it was suggested that P immobilised in the stem is reallocated to other plant organs as the plant ages (Crous et al., 2019). The ability to store P in the stems is an important trait also observed for some members of the *Proteaceae* from south-western Australia (Dell et al., 1987; Jeschke and Pate, 1995), and it was suggested that this accumulation pattern might be a mechanism to prevent leaf P toxicity in P-sensitive species (Shane et al., 2004). Seasonal remobilisation of P to the stems is also a strategy to store P in temperate forests species (Netzer et al., 2018, 2017). In natural stands of the perennial Proteaceae *Banksia prionotes* stem P accumulation is seasonally altered, with re-translocation of P accumulated during winter for summer flushing (Parks et al., 2000). Similar seasonal dynamics can be expected for P-fertiliser eucalypts.

Our results, however, showed that under low P, and except for *E. acmenoides*, seedlings of the studied eucalypt species had just slightly higher P concentrations in leaves than in the stems, indicating preferential P allocation to photosynthetic processes (Sardans and Peñuelas, 2004). However, variation among species and tissues suggests that eucalypts may adopt different mechanisms to improve P acquisition and accumulation under low soil availability. For example, roots of *E. acmenoides* had similar P concentrations when growing on low and sufficient P. But, leaves and stem of plants on sufficient P had higher concentrations than at low P. These results corroborate other studies with eucalypts and suggest that the relatively constant P concentrations in roots may exert a “buffering” control over translocation to the shoots, ensuring their demands (Mulligan, 1988). The lack of correlation between plant P concentration and biomass production suggests that greater P-uptake efficiency does not necessarily lead to higher plant growth and that plant P concentrations cannot be solely considered an indicator of the responsiveness of young eucalypts to P supply, supporting our previous results (Bulgarelli et al., 2019). In addition, observed in other crops (White et al., 2005; White and Hammond, 2008), our results show that selection for greater PUE does not appear to be an effective strategy for selecting eucalypt species to be cultivated on soils with low P availability. Only *E. acmenoides* presented significant differences between plants grown in low and sufficient soil P availability. Moreover, given the negative correlations between plant biomass production and P concentrations in *E. acmenoides* under low P availability, it is evident that low P-fertiliser inputs can be used for this species. Although it cannot be totally excluded the possibility that at sufficient P and for *E. acmenoides* other nutrients besides P could be restricting a higher growth.

Although only a small part of plant P is used for photosynthesis (Turnbull et al., 2007), this nutrient is essential for the proper functioning of the photosynthetic apparatus (Hammond and White, 2008). The electron transport in the photosystem I is affected by P deficiency and, consequently, impairs ATP production and reduces CO_2_ assimilation (Walker et al., 2014; Carstensen et al., 2018). Although low soil P availability frequently decreases the photosynthetic capacity of tree species (Bloomfield et al., 2014), here the low P availability did not affect the photosynthetic rate of the five studied eucalypt species. Nevertheless, *A*/*C*i curves showed that low P availability increased the *V*_cmax_, *J*_max_ and TPU in *E. tereticornis.* Because the stomatal conductance (*g*_*s*_) was not affected by low P, the observed photosynthetic response of *E. tereticornis* indicates that the efficiency of the biochemical reactions in the Calvin cycle led to a higher photosynthetic performance in this species when compared to the others.

Supporting the results of Bahar et al. (2018) and Bulgarelli et al. (2019), we observed that the eucalypt species with higher biomass production did not necessarily present the highest photosynthetic rates. Although biomass production derived from CO_2_ assimilation is only one of many factors influencing plant growth, other aspects, such as sucrose synthesis and transport, respiration and C partitioning, also significantly impact biomass production (Hofius and Börnke, 2007). P deficiency appears to affect photosynthetic rate by reducing ribulose bisphosphate (RuBP) regeneration, particularly the initial activity of ribulose-5-phosphate kinase, and by enhancing the flux of C into starch biosynthesis (Fredeen et al., 1990). In our study, while the ribulose-5P, an intermediate of Calvin-cycle, increased in leaves of *E. acmenoides*, it decreased in the other species grown under low P. The increase of ribulose-5P contents in *E. acmenoides* suggests a reduction in Calvin-cycle activity, which is supported by the low photosynthetic rate and the low PPUE of this species. Thus, it seems that low P availability may diminish RuBP regeneration in *E. acmenoides*. On the other hand, *E. tereticornis* increased PPUE under low P, indicating that this species may reduce the allocation of P into different foliar P fractions (e.g., nucleic acids, membrane lipids, and metabolites such as sugar phosphates) and prioritising metabolic-P (Hidaka and Kitayama, 2011; Lambers et al., 2012).

Plant P status has multiple effects in almost all aspects of plant metabolism, and there is insufficient knowledge on the effects of P deficiency on metabolite levels in evergreen trees (Warren, 2011; Netzer et al., 2018; Yang et al., 2020). In the present study, the hierarchical cluster analysis of primary metabolites from leaves differentiated *E. acmenoides* from the other species. At low P, fructose-6P levels significantly increased in leaves of *E. globulus* and *E. acmenoides*, unlike observed by Warren (2011) in seedlings of *E. globulus*. The low levels of fructose-6P in leaves of *E. tereticornis* may be related to its higher PPUE under low P, as previously reported in other eucalypts species (Warren, 2011; Bulgarelli et al., 2019). Thus, it seems that eucalypts may have distinct responses to low P availability, deserving a more careful investigation. The possibility of differential P-demands among eucalypt species cannot be excluded, leading to metabolic variations to enhance cellular P-use efficiency. The levels of most phosphorylated metabolites, such as glucose-6P and fructose-6P, showed enhanced turnover or reductions, which could be strategic to buffer cytosolic phosphate, either by releasing P from sugars-P or by reducing sugar-P synthesis (Veneklaas et al., 2012).

Stem metabolome data also separated the response of *C. maculata* and *E tereticornis* from that of *E. acmenoides* and *E. grandis*. The most significant differences were observed between *C. maculata* and *E. grandis*, which showed opposite trends in the levels of some metabolites to low P. Overall, the di- and trisaccharides, gentiobiose and raffinose, were significantly altered under low P availability, especially in the stem of both *C. maculata* and *E. grandis*. The accumulation of these saccharides under P-limitation can be a metabolic strategy to reduce P consumption in the phosphorylation of sugars (Nanamori et al., 2004; Huang et al., 2008). However, the low inorganic P pools, shown by a sharp reduction of phosphoric acid in all organs of *E. grandis*, the significant decrease of P concentration and the increased levels of glucose-6P and fructose-6P, indicate that di and trisaccharides accumulation seems not to be the preferential metabolic strategy in this species to cope with low P availability (Huang et al., 2008). These findings suggest that tree species that grow well and have low P concentrations when growing at low soil P availability can reduce the demand for metabolic P (Hidaka and Kitayama, 2013), as we observed here with *E. acmenoides.*

At low P, the sucrose content increased in the leaves but decreased in the roots of *E. tereticornis*. The root biomass also decreased. P shortage has the potential to initiate sugar signalling cascades that down-regulate several genes involved in plant responses to low P availability (Li et al., 2021); in this sense, the loading of sucrose to the phloem can reallocate C resources to the roots, which in turn may increase their growth (Hermans et al., 2006; Devaiah et al., 2009; Hammond and White, 2011). This metabolic adjustment for low P seems not to be the case for *E. tereticornis*, as sucrose was kept low in roots, and the root biomass did not increase. Consequently, the relatively small root system costs for P acquisition at low P soil since this species decreased the nutrient concentration in roots and stem.

The amino acid levels of *E. grandis* leaves were significantly affected by soil P availability in our study, unlike previously reported by Warren (2011). The foliar levels of isoleucine, leucine, methionine, alanine and homoserine, some intermediates of the fatty acid biosynthetic pathway, showed a small or non-significant effect of P supply. However, they increased in the roots of *E. globulus.* We consider three hypotheses to explain these results: (i) P shortage may lead to a general increase in amino acids due to an inhibition of protein synthesis or increased protein degradation (Rufty et al., 1990; Morcuende et al., 2007; Pant et al., 2015a,b), (ii) the C skeleton of amino acids can be used for energy production under nutrient limitation (Rasmusson and Møller, 2011), (iii) the ability of some plants to recapture exuded amino acids from the apoplast or directly from the rhizosphere (Warren, 2006; Perchlik et al., 2014). Another explanation is that the increase of alanine, leucine and isoleucine levels in roots reflects higher demand for the fatty acid pathway under P limitation, which is consistent with the lipid remodelling under low P (Lambers et al., 2012, Lambers et al., 2015a,b; Yuzawa et al., 2012; Shimojima et al., 2013; Kuppusamy et al., 2014). However, this seems to not happen in *E globulus.*

During plant development, phospholipids are the dominant polar lipid fraction in plasma membranes, while the galactolipids (MGDG and DGDG) and sulfolipids (SQDG) are key components of plastidic membranes (Moellering and Benning, 2011; Okazaki et al., 2013). The replacement of membrane phospholipids by galactolipids and sulfolipids has been reported in different plant species as a strategy to release P to essential metabolic processes, such as synthesis of ATP and nucleic acids (Tjellström et al., 2008; Lambers et al., 2012; Okazaki et al., 2013; Shimojima et al., 2013; Wang et al., 2020). Interestingly, at low P, *E. acmenoides* plants increased the levels of galactolipids and sulfolipids in leaves more significantly than the other eucalypt species. Glycerolipid synthesis in the chloroplast has critical importance for proper thylakoid biogenesis and photosynthesis functioning (Kobayashi, 2016; Li and Yu, 2018), and thus, lipid remodelling may lead to reduced rates of photosynthesis under low-P conditions (Wang et al., 2020). The low photosynthetic rates, observed mainly in *E. acmenoides*, suggest significant variability related to photosynthetic capacity and lipid remodelling among eucalypt species for increasing P-use efficiency in soils with low P availability. *E. acmenoides* exhibited a TAG accumulation at low P, and as previously reported, DAG and TAG can function as C-storage pools at low P conditions (Pant et al., 2015a,b). Additionally, TAG accumulation in response to P limitation is a well-known phenotype for green algae (Weers and Gulati, 1997; Lynn et al., 2000; Gong et al., 2013; Iwai et al., 2014), but so far in plants has only been reported for cell cultures of black mustard (Fife et al., 1990). Hence, these results provide a new perspective, indicating that TAGs (e.g., TAG:46) may be a metabolic marker in eucalypt plants grown under low soil P availability. Such information might be used for breeding purposes.

Based on metabolic and lipidomic profiles, we revealed several metabolites and their levels in leaves, stems and roots in five eucalyptus species. Although these organs contain many common metabolites, their levels were different. The concentrations of many primary metabolites, particularly sugars, organic acids, amino acids, and lipids, showed wide variability among eucalypt species that grew in soils with low P availability. In all organs, were observed a clear separation of *E. grandis* from the other species, thus indicating that it has a distinct metabolic response to low P. *E. grandis* was the only species to present significant differences in P concentration in response to soil P availability and in turn, to present low photosynthetic rate. Overall, this separation was characterised by a change in di- and trisaccharides, principally in roots and stem. As commented above, the abundance of saccharides and a significant reduction in the levels of phosphorylated metabolites suggest the enhancement of internal P utilisation at low P conditions (Ganie et al., 2015; Müller et al., 2015). Interestingly, for stems and roots, hexadecanoic, octadecanoic, decanoic, and nonanoic acid, all intermediates of the fatty acid pathway, were responsible for grouping *C. maculata*, *E. tereticornis* and *E. acmenoides* in the PCA analysis, thus indicating changes in lipid metabolism in the lipids profiles (Okazaki et al., 2013). This metabolic correlation between lipids and primary metabolites in response to P availability may be helpful to investigate membrane lipid remodelling and identify new critical metabolites with a role in the ability of eucalypt species to cope with low P availability.

In conclusion, the eucalypts species investigated here had different growth responses to low P. They also differed regarding biochemical mechanisms, which may improve the use of P. These varied responses are probably related to the genetic background since it is known that the Australian continent, the origin centre of eucalyptus, has soils with low P availability (Vitousek et al., 2010; Viscarra Rossel and Bui, 2016). This knowledge could be the basis for planning interspecific crosses in breeding programs, aiming to (i) improve crop yield under low P availability or low P inputs and (ii) develop new molecular breeding strategies to increase the production of P-efficient crops. Considering estimates that P rock reserves may be exhausted in the following decades (Alewell et al., 2020) and that P is strongly adsorbed by the soil particles limiting crop production, research aiming to increase P use efficiency becomes a sustainable and necessary strategy.

## Data availability statement

The original contributions presented in this study are included in the article/Supplementary material, further inquiries can be directed to the corresponding authors.

## Author contributions

PM and SA designed the research. FS, RB, and UM performed the experiments and the laboratory analyses. FS and UM performed the data analysis and the statistics. CC and UM assisted FS in the metabolome and lipidome analyses. FS, CC, SA, and PM wrote the manuscript. All authors read and approved the final manuscript.
