# Supplementary material for: Low phosphorus induces differential metabolic responses in eucalyptus species improving nutrient use efficiency
Source: Front Plant Sci. 2022 Sep 15;13:989827. doi: 10.3389/fpls.2022.989827 (PMC9520260; doi:10.3389/fpls.2022.989827)
Supplement: Supplementary file 1 [file Table_1.DOCX]

**Table S1**. Chemical characteristics of the substrate (soil/sand mixture) used to grow eucalypt plants under low P and sufficient P conditions.

| Phosphorus | **OM** | **pH CaCl_2_** | **P_res_** | **K** | **Ca** | **Mg** | **H+Al** | **CEC** | **BS** | **S** | **Fe** | **Mn** | **Cu** | **Zn** | **B** |
| --- | --- | --- | --- | --- | --- | --- | --- | --- | --- | --- | --- | --- | --- | --- | --- |
|  | g dm^-3^ |  | mg dm^-3^ | mmol_c_ dm^-3^ | | | | | % | mg dm^-3^ | | | | | |
| Low P | 5.0 | 6.3 | 4.5 | 0.8 | 9.4 | 5.9 | 11.3 | 27.6 | 58.6 | 40.5 | 3.6 | 1.4 | 0.2 | 4.1 | 0.1 |
| Sufficient P | 5.0 | 6.1 | 10.8 | 1.4 | 8.5 | 6.3 | 11.8 | 27.9 | 57.5 | 44.7 | 3.7 | 1.7 | 0.2 | 4.3 | 0.1 |

OM: organic matter, CEC: Cation Exchange Capacity; BS: base saturation
