# Supplementary material for: Low phosphorus induces differential metabolic responses in eucalyptus species improving nutrient use efficiency
Source: Front Plant Sci. 2022 Sep 15;13:989827. doi: 10.3389/fpls.2022.989827 (PMC9520260; doi:10.3389/fpls.2022.989827)
Supplement: Supplementary file 2 [file Table_2.docx]

Table S2. Photosynthetic assimilation rate (A), leaf P content (P leaf), photosynthetic P-use efficiency (PPUE) and P utilization efficiency (PU_t_E). The parameters of P use efficiency were calculated according to Hammond et al. (2009) and Hidaka & Kitayama (2013).

|  |  |  |  |  |
| --- | --- | --- | --- | --- |
| **Eucalypts** | **A_low P_ (µmol CO_2_ m^-2^ s^-1^)** | **A_suf P_ (µmol CO_2_ m^-2^ s^-1^)** | **P leaf_low P_ (g leaf^-1^)** | **P leaf_suf P_ (g leaf^-1^)** |
| *E. acmenoides* | 7.11 | 8.59 | 0.00445 | 0.00460 |
| *E. globulus* | 6.44 | 6.50 | 0.00316 | 0.00487 |
| *E. grandis* | 6.34 | 6.01 | 0.00359 | 0.00533 |
| *C. maculata* | 7.76 | 7.61 | 0.00495 | 0.00795 |
| *E. tereticornis* | 11.08 | 10.77 | 0.00503 | 0.00458 |
|  |  |  |  |  |
|  |  |  |  |  |
|  | **PPUE_low P_ (µmol CO_2_ mol P^-1^ s^-1^)** | **PPUE_suf P_ (µmol CO_2_ mol P^-1^ s^-1^)** | **PU_t_E (g DM g^-1^ P)** |  |
| *E. acmenoides* | 1599.4 | 1868.8 | 13093.77 |  |
| *E. globulus* | 2037.9 | 1335.6 | 1133.01 |  |
| *E. grandis* | 1766.4 | 1126.0 | 490.07 |  |
| *C. maculata* | 1567.8 | 956.6 | 382.64 |  |
| *E. tereticornis* | 2204.0 | 2351.6 | 558.96 |  |
|  |  |  |  |  |
