# Supplementary material for: Low phosphorus induces differential metabolic responses in eucalyptus species improving nutrient use efficiency
Source: Front Plant Sci. 2022 Sep 15;13:989827. doi: 10.3389/fpls.2022.989827 (PMC9520260; doi:10.3389/fpls.2022.989827)
Supplement: Supplementary file 3 [file Table_3.docx]

Table S3. Photosynthesis related parameters of five eucalypt species cultivated in soil with low (Low P) or sufficient (Suf P) availability of P. Means followed by the same letter indicated no statistical difference by the Skott Knott test (P < 0.05). Lower case letters compare the species at the same P availability and upper case letters compare the effect of P availability within each species.

*A,* CO_2_ assimilation rate; *g*_s,_  stomatal conductance; *g*_m_, mesophyll conductance to CO_2_; *R*_d_, leaf dark respiration in the light; *C*_i_, intercellular CO_2_ concentration; Trmmol, transpiration rate*; photochemical (qP) and non-photochemical (qN) quenching*; ETR, electron transport rate; *Vcmax_C_i_*, maximum carboxylation velocity based on *C*_i_; *Jmax_Ci*, maximum capacity for electron transport rate based on *Ci* and TPU*,* triose-phosphate utilization. The Vcmax, Jmax, TPU, *R*_d_ and *g*_m_ was solved simultaneously using the whole A–Ci curve data points as described by Sharkey *et al*.(2007).
